# Seasonal and individual variation in the use of rail-associated food attractants by grizzly bears (*Ursus arctos*) in a national park

**DOI:** 10.1371/journal.pone.0175658

**Published:** 2017-05-24

**Authors:** Maureen H. Murray, Sarah Fassina, John B. Hopkins, Jesse Whittington, Colleen C. St. Clair

**Affiliations:** 1 Department of Biological Sciences, University of Alberta, Edmonton, Alberta, Canada; 2 Banff National Park, Banff, Alberta, Canada; University of Sydney, AUSTRALIA

## Abstract

Similar to vehicles on roadways, trains frequently kill wildlife via collisions along railways. Despite the prevalence of this mortality worldwide, little is known about the relative importance of wildlife attractants associated with railways, including spilled agricultural products, enhanced vegetation, invertebrates, and carcasses of rail-killed ungulates. We assessed the relative importance of several railway attractants to a provincially-threatened population of grizzly bears (*Ursus arctos*) in Banff and Yoho National Parks, Canada, for which rail-caused mortality has increased in recent decades without known cause. We examined the relationship between the use of the railway and diet by fitting 21 grizzly bears with GPS collars in 2011–2013 and measuring the stable isotope values (*δ*^15^N, *δ*^34^S) derived from their hair. We also examined the importance of rail-associated foods to grizzly bears by analyzing 230 grizzly bear scats collected from May through October in 2012–2014, some of which could be attributed to GPS-collared bears. Among the 21 collared bears, 17 used the rail rarely (<9% of the days they were monitored), and only four bears (which included the three smallest bears and the largest bear in our sample) used the rail frequently (>20% of their monitored days). We found no significant relationships between *δ*^15^N and *δ*^34^S values measured from the hair of grizzlies and their frequency of rail use. Instead, *δ*^15^N increased with body mass, especially for male bears, suggesting large males consumed more animal protein during hair growth. All four bears that used the railway frequently produced scats containing grain. Almost half the scats (43%) collected within 150 m of the railway contained grain compared to only 7% of scats found >150 m from the railway. Scats deposited near the rail were also more likely to contain grain in the fall (85% of scats) compared to summer (14%) and spring (17%), and those containing grain were more diverse in their contents (6.8 ± 2.2 species vs. 4.9 ± 1.6, P < 0.001). Lastly, scats collected near the rail were more likely to contain ungulate hair and ant remains, especially in the summer. Our results support local management knowledge that some bears in the region use the railway to forage and supplement their diets with spilled grain, but that individual use of the railway and associated foods were highly variable. We suggest that managers continue to reduce the risk of bears being killed by trains by reactively removing grain and ungulate carcasses from the railway, reducing the amount of grain spilled by trains, and target mitigation to the specific individuals and locations that attract recurrent rail-based foraging.

## Introduction

The negative effects of roads on wildlife movement and survival are well-documented (reviewed in [1,2]), but few studies have examined the effects of railways on wildlife. Like roads, railways are efficient transportation systems [3] that degrade or fragment wildlife habitat (e.g. [4]), impede or facilitate animal movements [5,6], and are sites where wildlife mortality occurs through train-wildlife collisions (reviewed in [7,8]. Although traffic volume is lower on railways than roads, the per-vehicle rate of collisions and mortality can be much higher for trains because they cannot steer around animals on tracks, are more massive and take longer to stop [9], and often occur in less-disturbed landscapes [10]. Moreover, the lower risk of human injury in train-wildlife collisions lessens public demand for mitigation that result from wildlife collisions on roads. These factors may explain why several authors have found that rates of wildlife mortality from railways can be higher than on adjacent roads [11–14]. Just as vehicle collisions on roads have compromised the persistence of threatened populations of several species, including Florida panthers (*Puma concolor coryi*), key deer (*Odocoileus virginianus clavium*; [15]) and Blanding’s turtles (*Emydoidea blandingii*; [16]), train collisions with wildlife on railways can cause population declines [17]. Understanding the behaviour of animals that use the railway, and may thus have a higher risk of train collisions, could help mitigate train-wildlife collisions worldwide.

Reducing wildlife mortalities by trains and other vehicles is a major focus for wildlife managers in Banff and Yoho National Parks, where the mainline of the Canadian Pacific Railway shares valley bottoms and mountain passes with parallel infrastructure that includes the TransCanada Highway and secondary roads (reviewed by [18,19]). Since 1982 (when record-keeping was standardized) at least 1256 large mammals were killed by trains in Banff and Yoho, including five species of ungulates and four carnivore species (summarized by Gilhooly et al. in preparation). Although human-induced mortality has been the leading cause of grizzly bear (*Ursus arctos*) deaths (*N* = 19) in the region for several decades [20], train collisions have increased since 1998 and have become the largest current source of mortality for grizzlies [21] with a rate that cannot likely be sustained by the local population of around 60 animals [22]. Increased rail use by bears, and hence mortality, may have occurred because of the relative value of one or more rail-associated benefits for bears, including spilled agricultural products (hereafter, grain; e.g., [23]), enhancement of adjacent vegetation [10], and higher travel efficiency [24]. One or more of these factors could attract bears directly or indirectly by attracting other species that bears predate or scavenge. In Alberta, the protein subsidies available to bears on the railway via train-killed ungulates may be especially important, as grizzly bears appear to be limited by foods that are high in fat and animal protein [25,26].

Wildlife researchers could assess the relative attraction of populations to transportation corridors and other human use areas by measuring the diet, movement, and habitat selection of individuals. Equally important to understanding the population-level extent and effects of this attraction, researchers must investigate the causes and consequences of diet variation among individuals (e.g., by age and sex classes) and through time (e.g., season; [27]). Wildlife diet is often assessed via analysis of scat contents, which can be especially informative for wide-ranging, omnivorous species such as canids [28] and bears (e.g., [29]). Unfortunately, fecal analysis is limited in its ability to provide accurate information about the digestible component of omnivore diets. Stable isotope analysis can complement fecal analysis by providing information about the assimilated diets of consumers, including omnivorous bears [30]. This is typically achieved by assessing the variation of stable isotope ratios in the tissues of consumers and their foods and using these measurements to estimate the diets of populations and individuals [31,32].

Stable isotopes are also useful for detecting conflict-prone individuals [33–36]. For instance, hair from black bears (*Ursus americanus*) that foraged for meat-rich, human foods had higher nitrogen isotope ratios (^15^N/^14^N, expressed as *δ*^15^N values) than hair from bears that primarily foraged for plants [35,37]. Similarly, the hair from grizzly bears in Banff that were killed on or captured near the railway had higher *δ*^15^N and *δ*^34^S values (^34^S/^32^S) than bears captured away from rail [38]. Enrichment of ^15^N in grizzly bear hair suggested that individuals using the railway in Banff consumed more meat, likely from train-killed ungulates [30], whereas relatively high *δ*^34^S values for bear hair indicated that bears ingested sulfur pellets directly when foraging for grains or indirectly from the rail (i.e., from plants that grew along the rail in sulfur-enriched soil or from animals that consumed those plants).

Combining information about diet components with space use makes it possible to reveal relationships among movement rates, habitat selection, diet, trophic position, and animal health (e.g., [39,40]). We used three techniques to assess the diet (scat and stable isotope analyses) and rail use (GPS technology with VHF radio-telemetry) of grizzly bears captured along the railway in Banff and Yoho National Parks. We hypothesized that bears are attracted to the railway to forage for rail-associated foods. Our hypothesis predicted that rail use by individual bears would correlate with higher levels of *δ*^15^N and *δ*^34^S in bear hair, as ^15^N and ^34^S are enriched in the tissues of individuals that forage for more animal protein [41] and spilled sulfur pellets on the rail [38], respectively. We also expected that the relationships between rail use and diet would depend on bear age and sex because large adult males often have higher protein requirements [42,43]. Lastly, if bears are attracted to the railway to forage for rail-associated foods, then scats collected near the rail would be more likely to contain grain, plants associated with open habitats and edges, hair from ungulates that might have been killed on the rail, and invertebrates attracted to the higher temperatures and grain on the railway—all of which we expected to be more prevalent in spring and fall when bears have higher energetic demands and fewer rich alternate foods.

## Materials and methods

### Study area

We investigated the diets and railway use of grizzly bears in Banff and Yoho National Parks, Alberta, Canada, located in the Canadian Rocky Mountains (51.2°N, 115.5°W; 6,641 km²). Banff and Yoho are comprised of montane, subalpine, and alpine ecoregions and contain several mountain ranges where elevation varies from 1000 to 3500 m. Grizzly bears mainly occupy the montane and subalpine ecoregions, which contain important plant-based foods for bears, including buffaloberries (*Shepherdia Canadensis*), bearberries (*Arctostaphylos uva-ursi*), sweet vetch roots (*Hedysarum* spp.), graminoids, and horsetails (*Equisetum* spp.; [44]). Banff also contains several ungulate species, including mule deer (*Odocoileus hemionus*), white-tailed deer (*O*. *virginianus*), moose (*Alces alces*), and elk (*Cervus canadensis*). Human development has expanded in Banff, attracting up to 4 million visitors annually [45]. Banff is bisected by the TransCanada highway and the Canada Pacific Railway, both of which have been important sources of mortality for many species of wildlife, including grizzly bears, other carnivores such as black bears and wolves, and ungulates [46]. Along the Canada Pacific Railway, tall vegetation is cleared up to 30 m on either side of the railway to increase visibility for engineers and to reduce potential for fire. Clearing vegetation in such a manner promotes growth of herbaceous vegetation that can adapt to recent disturbances, such as clover (*Trifolium* spp.), dandelion (*Taraxacum officinale*), horsetail (*Equisetum* spp.), and other forbs. These species are preferred by grizzly bears in the region [44].

### Bear collaring and hair collection

Parks Canada personnel captured grizzly bears from 2011–2013 using free-range darting and culvert traps. Captures followed protocols approved by Parks Canada Animal Care Committee (Parks Canada Research Collection Permit LL- 2012–10975) and are described by Hopkins et al. [38] and Whittington et al. [47]. Parks Canada weighed, sexed, and fitted each bear with a GPS collar (GPS-Plus from Vectronic Aerospace GmbH, Berlin, Germany; Tellus from Followit Wildlife Lindesberg, Sweden) and collected a clump of guard hairs for stable isotope analysis. Hair sample preparation and analysis followed the methods described in Hopkins et al. [38]. Parks Canada monitored all GPS-collared bears in this study for an average of eight months (mean = 238 ± 156 SD days).

### Rail use

We estimated rail use by GPS-collared bears using fixes generated every 2 hours (either via the programmed schedule or sampled from a more frequent rate). We first estimated overlap between the railway and 95% kernel density home ranges and then calculated rail use as the proportion of days a bear spent at least four consecutive hours within 30 m of the railway using a Geographic Information System (Arcmap 10.1, Redlands, CA). We used a 30 m buffer of 15 m on either side of the rail to accommodate GPS collar error. We measured daily rail use rather than habitat selection (for the rail) to reduce the effects of autocorrelation between successive GPS locations. We also measured whether bears used portions of the rail that are associated with higher rates of rail-killed ungulates by calculating the density of ungulate mortalities within 400 m (corresponding to the average 95% utilization distribution of study bears of 489 km^2^) of each bear GPS location that was within 30 m of the railway. Parks Canada Agency provided spatial data for rail location and ungulate mortalities from 1982 to 2014.

### Scat collection and analysis

**Fig 1:**
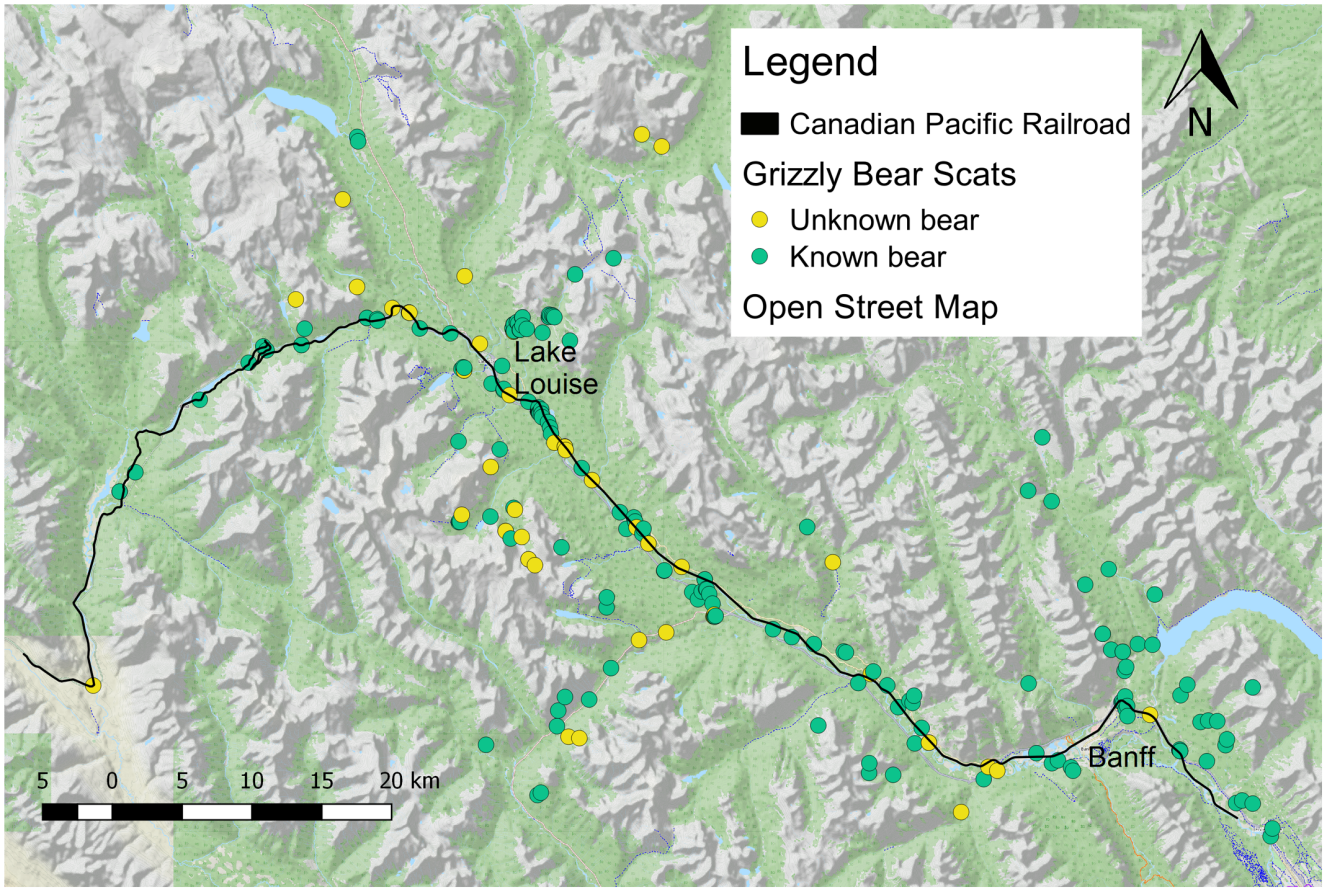
Map of study area. Map of the Canadian Rocky Mountains showing the locations of grizzly bear scats collected opportunistically (blue) or at clusters of bear GPS locations (yellow) in Banff and Yoho National Parks. Background map OpenStreetMap contributors available under the Open Database License.

We examined which types of foods were consumed by bears along the railway by collecting grizzly bear scat throughout a 2,383 km² area in Banff (Fig 1). We conducted our survey in spring (May and June), summer (July and August) and fall (September and October) in 2012–2014. We collected scats via targeted searches around clusters of locations where GPS-collared bears had spent at least four hours, opportunistically while collecting vegetation samples at 18 sampling sites associated with the rail, and at several backcountry sites as part of a concurrent study on bear attractants (Pollock et al. in preparation). Scat samples were collected under Parks Canada research collection permits LL-2012-10975 and YNP-2012-11155. We collected scats up to 25 km from the rail at elevations ranging from 1114 to 2358 m. We examined the relationship between proximity to the rail and bear diet composition in two ways. Firstly, we used linear regression to quantify changes in scat components with distance to the rail. Secondly, we quantified the importance of spilled grain, vegetation, and animal prey for bears using the rail by comparing the contents of scats collected near and far from the rail. Many scats (37%) were found within 150 m from the rail, after which there was sharp decrease in scat occurrence (8% of scats found between 150 m and 300 m). Based on this spatial distribution, we considered a scat as *near* the rail if collected within 150 m of the rail and *far* if collected at distances >150 m from the rail.

Of the 308 bear scats we collected, we assigned 230 to grizzly bears based on one or more of the following criteria: (a) the scat was collected near a cluster of grizzly GPS locations (n = 178); (b) the scat was collected opportunistically at a site where other grizzly bear sign was recorded (e.g. digging and tracks; n = 39); or (c) the scat was collected opportunistically and had a similar dry mass (mean ± S.D.: 221.2 ± 137.4 g; n = 13) as grizzly scats at other cluster sites (209 ± 145.2 g) that was heavier than the scats we assigned (via similar clues) to black bears (*Ursus americanus*; 89.3 ± 15.7 g; n = 78).

Once collected, scats were dried for at least 24 hours and frozen at -20°C. We separated undigested food remnants from fecal material by rehydrating the scats and washing them through a 4 mm sieve and then a finer 1 mm sieve. Once separated, we examined food remnants using dissecting microscopes. We identified undigested components of bear foods via the morphology of seeds and husks (for grain and berries), plant parts (including leaves, stems, and roots), and hair (for ungulates) using descriptive and dichotomous keys [48].

For each scat, contents were mixed and subsampled into a 1L pan of water for examination [49] and we visually estimated the proportion of each scat comprised by each food item to calculate the *percent volume*. We also calculated the proportion of scats containing each item (*frequency of occurrence*). For both measures, we included categories for unidentified material. We included sulfur pellets (which only occurred on the rail after leaking from hopper cars) to estimate the distance from the rail at which other rail-originating products may have been deposited by bears.

### Data analysis

We compared the frequencies and volumes of three categories of scat contents as a function of their distance from the rail (both as a continuous measure and as binary categories of being ≤ or >150 m) and season (Spring, Summer, Winter). The three categories identified grain, plants associated with disturbed landscapes and/or edges, and animals associated with railways (invertebrates in track ballast and ungulates that might have been struck and then scavenged on the railway). We used linear regression to test whether the occurrence or volume of grain, vegetation, or animals decreased with log-transformed distance to rail to account for the right-skewed distribution of scat distances to the railway. We examined potential differences in the frequency of occurrence using contingency tables and tests of independence with years as replicates. We used replicated Barnard’s unconditional exact tests because they have higher statistical power suitable for small sample sizes [50]. We tested for differences in percent volume using t tests. For both types of tests, we accounted for multiple comparisons using a Sidak-adjusted p-value [51]. We also tested whether bears appeared to specialize on grain or forage opportunistically by comparing the average number of species per scat across scats that did or did not contain grain. Lastly, we used linear regression to test whether *δ*^15^N or *δ*^34^S values from hair were positively correlated with rail use, as results from a previous study suggest that hair from bears that were killed or captured on the rail have higher *δ*^15^N (from consuming train-killed ungulates) and *δ*^34^S values (from ingesting sulfur pellets) than conspecifics sampled throughout the park [38].

## Results

We collected and analyzed hair samples from 21 bears fitted with GPS collars in 2009–2013, including 9 adult males, 8 adult females, 3 subadult males, and 3 subadult females (Table 1). We collected GPS data from bears for an average of 11.0 ± 7.5 (range: 2–28) months, which resulted in 2,162 ± 1,507 (range: 340–6449) 2-hour locations per bear. We also analyzed 230 grizzly bear scats collected in August—October 2012 (n = 74) and May—October 2013 (n = 115) and 2014 (n = 41). Of these, we collected 85 (37%) ≤150 m from the rail and 145 (63%) >150 m from the rail. We were able to assign scats to 18 collared grizzly bears (10.0 ± 9.8 scats per bear, range = 2–41).

**Table 1:** Summary information for 21 grizzly bears fitted with GPS collars to quantify use of the Canadian Pacific Railway in Banff and Yoho National Parks in Alberta, Canada.

Bear ID	Sex	Age	Mass (kg)	Percent days on rail	*δ*^34^S	*δ*^15^N	Number of associated scats
64	Female	Adult	95	4.7	5.4	3.7	4
72	Female	Adult	83	2.7	11.2	4.2	18
122	Male	Adult	210	23.0	9	7	5
125	Male	Adult	108	1.2	7.5	3.3	0
126	Male	Adult	110	6.4	9	5	25
128	Male	Subadult	32	42.7	6.7	3.4	41
130	Female	Adult	110	7.3	5	4.4	13
131	Female	Adult	105	0	3.7	3.5	16
132	Male	Adult	90	0.7	4.6	5	0
133	Female	Adult	78	1.6	11.1	3.2	4
134	Male	Adult	190	6.7	8.7	4.9	6
135	Female	Adult	118	0	3.4	4	10
136	Male	Adult	180	3.4	7	6.4	4
138	Female	Adult	92	0	10.1	3	6
140	Male	Adult	111	0	11	3.5	0
141	Male	Adult	134	6.5	10.3	3.7	2
142	Female	Subadult	54	20.4	11.4	4.3	5
143	Female	Subadult	50	8.4	10.9	4	3
144	Male	Subadult	56	6.5	7	4.1	8
148	Female	Subadult	61	5.4	6.9	4.2	8
149	Male	Subadult	80	20	7.5	3.3	5

All of the 21 GPS-collared bears’ home ranges included the railway and, of these, 19 grizzly bears used the railway during at least one of the days they were collared. Most bears that used the rail (15 of 19) rarely did so (i.e. were within 15 m of the rail for <10% of days monitored, mean ± S.D. = 3.7 ± 3.3% of days monitored, range = 0–9.3%). However, four bears used the rail more frequently than conspecifics (i.e. near the rail for ≥20% of days monitored; 26.5 ± 10.9%, range = 20.1–54.0%).

**Fig 2:**
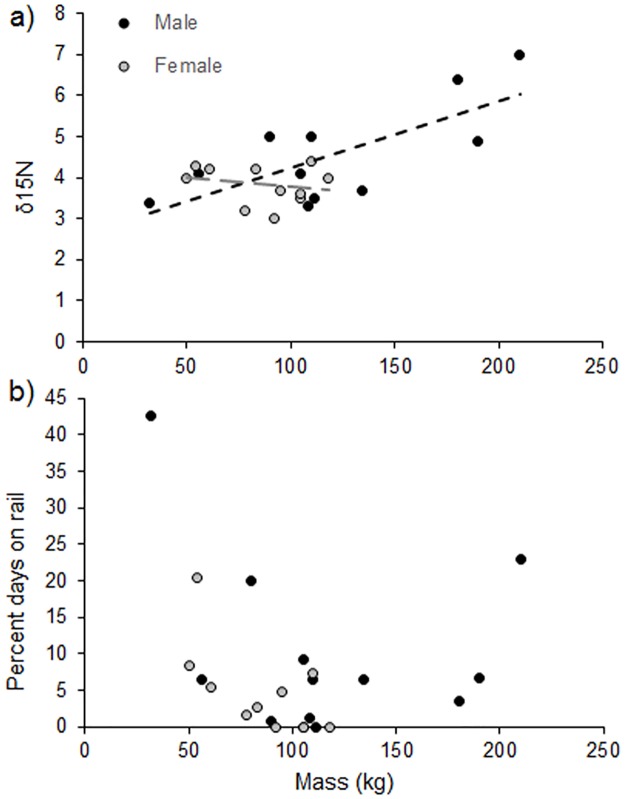
Bear mass and diet. Relationships between bear mass at time of capture and (a) *δ*^15^N values derived from bear hair, and (b) use of the rail for GPS-collared male and female grizzly bears in Banff National Park. Dashed lines indicate linear regression lines.

Contrary to our predictions, bear hair *δ*^34^S and *δ*^15^N values did not correlate with the proportion of days bears spent near the rail (*δ*^34^S: r² = 0.00049, F(1, 21) = 0.010, P = 0.92; *δ*^15^N: r² = 0.029, F(1, 21) = 0.63, P = 0.44). It seems, however, that the relationship between *δ*^15^N and rail use was confounded by the mass, and possibly sex, of bears in Banff. We found that *δ*^15^N values increased with body size for male bears (r² = 0.44, F(1, 21) = 16.5, P < 0.01, Fig 2a) and rail use was highest for the largest and smallest bears (Fig 2b). Of the four bears that used the rail frequently, three bears were smaller than average (32–80 kg, 52.8 ± 20.3 kg; two subadult males and one subadult female) and one was the largest male captured (210 kg; average bear size = 99.81 ± 41.94 kg; Table 1).

Grain occurred more frequently in scats collected closer to the railway (Occurrence vs. log meters to rail: R² = 0.17, P < 0.0001), however percent volume of grain in scats did not change significantly with distance (R² = 0.06, P = 0.32). Grain was commonly found in scats collected near the rail (42.6 ± 24.1%) but rarely in scats found far from the rail (6.8 ± 2.8%, G = 41.3, df = 1, P < 0.001, Table 2). The most common types of grain found in scats (n = 37) were wheat and barley (n = 20; grouped because they have similar nutritive qualities and are difficult to distinguish), followed by canola seeds (n = 17), lentils (n = 14), chickpeas (n = 3), and soybeans (n = 1). Scats collected near the rail contained higher volumes of wheat, canola, or lentils, which were collectively found in 93% of scats containing grain (Near: 22.3 ± 11.9%, Far = 3.1 ± 5.6% of scat volume, G = 39.8, df = 1, P < 0.001). Scats containing grain contained more types of plants and other native foods (with grain: 6.8 ± 2.2 species/scat) than those without grain (4.9 ± 1.6 species/scat, t = 4.95, df = 43, P < 0.001). Scats containing grain and sulfur pellets (n = 24) were found up to 7.2 km away from the rail and 97% of scats containing sulfur pellets also contained grain. Rail proximity did not influence the frequency of occurrence for plants associated with edges or disturbances (R² ≤ 0.09, P ≥ 0.29; Near = 61.8 ± 3.5%, Far = 70.6 ± 11.0%; Wald = 1.64, df = 1, p = 0.28) and these plants did not occur at higher volumes near the rail (R² = 0.05, P = 0.46; Near: 25.6 ± 26.5, Far: 45.6 ± 9.0; t = 0.7, p = 0.34; Table 2). Ungulate hair and ant exoskeletons were more common in scats collected near than far from the rail (R² = 0.12, P = 0.06; Near: 48.5 ± 20.4, Far: 37.5 ± 4.4; Wald = 5.89, df = 1, P = 0.02, Table 2). Raw values of scat percent volumes can be found in S1 Table.

**Table 2:** Summary of frequency of occurrence (scats containing item / total scats x 100) and percent volume (both mean ± SD) of nine food types hypothesized to be targeted by bears in the vicinity of railways in scats collected near (< 150m) and far (>150m) from the railway in Banff National Park, Alberta, Canada. P values refer to replicated Barnard’s tests (frequency data) or t tests (volume data).

	Frequency of occurrence				Percent Volume			
Food Type	Overall	Near	Far	P	Overall	Near	Far	P
Agricultural products	14.7 ± 6.7	42.7 ± 23.8	6.8 ± 2.8	0.01	45.3 ± 26.1	57.4 ± 34.3	56.3 ± 32.7	0.79
*Wheat/barley*	17.0 ± 11.5	58.0 ± 29.0	9.6 ± 7.6	0.01	21.5 ± 17.1	23.0 ± 12.9	15.8 ± 11.2	0.21
*Canola seed*	13.7 ± 12.8	48.7 ± 42.3	8.7 ± 7.0	0.05	15.8 ± 15.6	17.0 ± 13.0	15.0 ± 1.2	0.57
*Lentil*	10.7 ± 12.4	41.5 ± 32.2	6.2 ± 7.8	0.01	29.8 ± 23.5	36.1 ± 19.3	14.9 ± 1.1	0.03
*Chickpea*	6.1 ± 9.9	17.9 ± 25.3	4.8 ± 7.4	0.74	50.6 ± 40.1	11.1 ± 10.5	50.5 ± 57.3	0.82
*Flax*	5.3 ± 7.2	14.3 ± 20.2	4.3 ± 5.2	0.12	0.5 ± 0	0.7 ± 0.9	0.5 ± 0.6	0.77
*Soybean*	1.6 ± 0.8	5.1 ± 2.8	-	0.01	32.2 ± 53.6	48.1 ± 65.1	-	0.01
*Pea*	0.5 ± 0.8	3.6 ± 5.1	-	0.01	8.1 ± 0	8.1 ± 0	-	0.01
Rail-associated plants	70.1 ± 5.0	61.8 ± 3.5	70.6 ± 11.0	0.41	44.4 ± 35.6	25.6 ± 26.5	45.6 ± 9.0	0.15
Dandelion (*Taraxacum* sp.)	24.8 ± 10.5	9.8 ± 3.8	25.0 ± 6.6	0.02	36.6 ± 34.8	13.2 ± 17.2	35.1 ± 8.6	0.04
Horsetail (*Equisetum* sp.)	19.6 ± 2.3	14.5 ± 10.4	20.5 ± 4.1	0.37	23.7 ± 32.2	5.9 ± 2.5	32.7 ± 8.6	0.01
Sweetvetch (*Hedysarum boreale*)	16.3 ± 3.6	22.1 ± 9.2	15.6 ± 4.6	0.21	35.6 ± 33.6	29.3 ± 34.3	35.2 ± 7.3	0.63
Clover (*Trifolium* sp.)	10.6 ± 8.5	7.8 ± 11.0	11.7 ± 9.1	0.55	49.5 ± 34.4	53.3 ± 12.1	41.3 ± 22.4	0.38
Rail-associated animals	37.8 ± 3.9	48.5 ± 20.4	37.5 ± 4.4	0.04	16.0 ± 19.2	16.7 ± 20.4	12.5 ± 13.0	0.73
Ants (*Formicidae* sp.)	29.4 ± 2.3	42.7 ± 21.8	27.3 ± 6.4	0.31	14.8 ± 15.0	12.0 ± 13.3	15.6 ± 15.5	0.42
Ungulates (*Ungulata* sp.)	10.4 ± 6.0	12.0 ± 2.8	5.0 ± 7.5	0.41	16.8 ± 28.9	10.7 ± 6.4	17.4 ± 30.4	0.78

**Fig 3:**
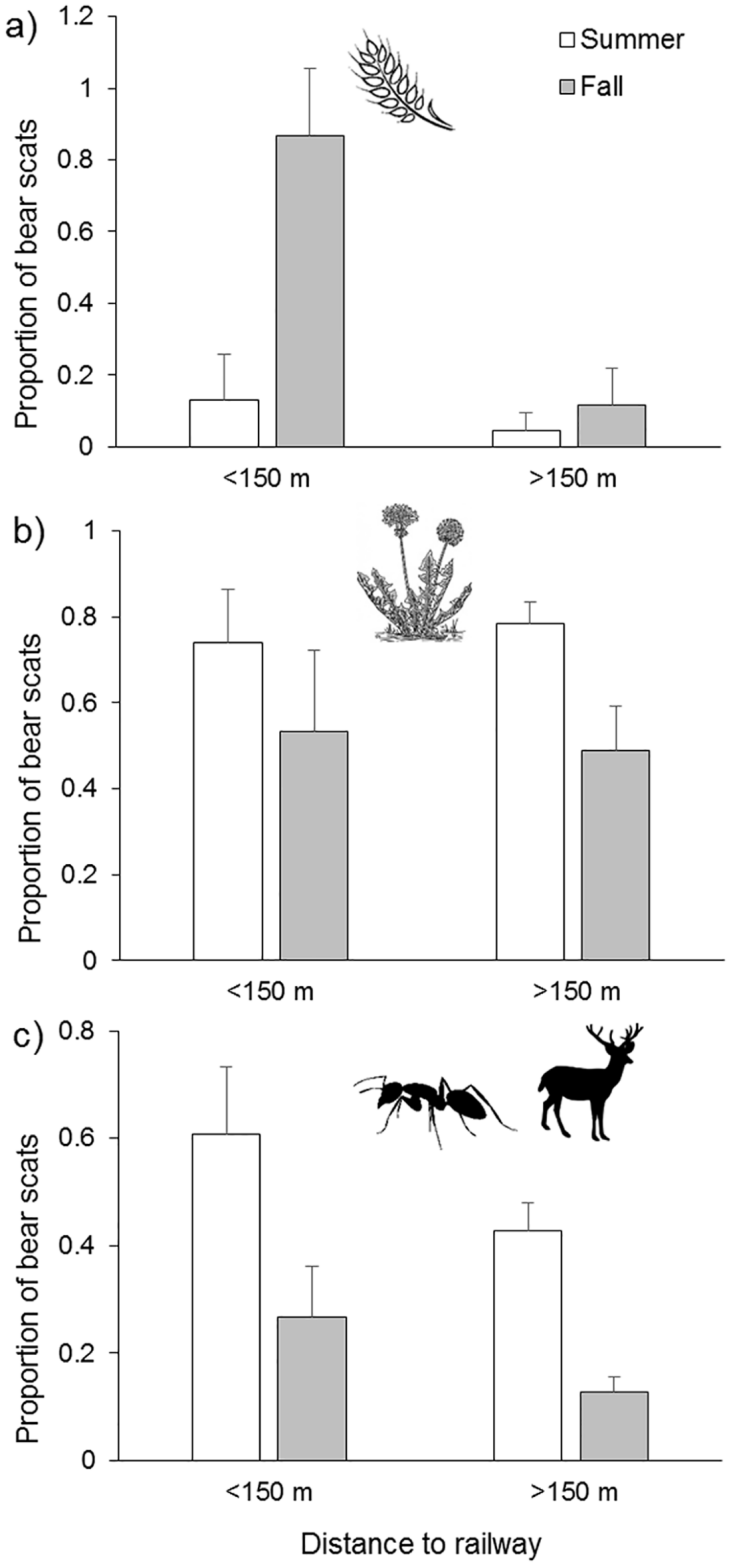
Scat contents near and far from the railway. The frequency of occurrence of (a) spilled grain, (b) plants associated with edges and disturbance (dandelion, clover, equisetum, sweet vetch) and (c) ungulate or ant remains in grizzly bear scats collected near (<150m) and far (>150m) from the railway in summer (July and August) and fall (September and October) in Banff National Park. Bars show standard deviation.

Near the rail, we identified grain in scats more frequently in the fall (87 ± 20% of scats, n = 90) than in the spring (17.4% of 28 scats; G = 35.1, df = 1, P < 0.05; spring 2013 only) and summer (13 ± 13%, n = 117; Wald = 3.03, df = 1, P = 0.002; Fig 3a). We found no seasonal differences in the presence of rail-associated plants in scats (Fall = 51 ± 19%, Summer = 72 ± 25%, Spring = 63%, G ≤ 9.5, df = 1, P ≥ 0.2; Fig 3b), but found more evidence of ungulates and ants in scats collected in the summer (Wald = 12.6, df = 1, P = 0.003; Fig 3c).

We collected at least five scats from 13 GPS-collared bears (14 ± 11 scats per bear), 10 of which used the rail at least once, and four of which used the rail frequently (Table 1). All four of these bears that use the rail frequently were associated with scats containing grain (average frequency of occurrence: 39.0 ± 7.7%) and sulfur pellets (18.8 ± 14.1%) and were the only bears associated with scats containing grain. We collected 41 scats (23% of all scats from known bears) from the smallest bear, who used the rail more frequently than the other study animals (43% of days monitored), consuming a variety of attractants (frequency of occurrence: grain = 34%, rail-associated vegetation = 59%, ungulates = 7%, ants = 14%) and appeared to consume large volumes of grain (percent volume: 72.0 ± 26.3%). Another small male (80 kg) consumed high volumes of rail-associated plants (100% of 5 scats, percent volume = 91.5 ± 32.7%) and no rail-associated animals. The largest male consumed ungulates (40% of 5 scats, percent volume = 10.2 ± 4.3%), no rail-associated vegetation, and used areas of the rail with higher densities of ungulate mortalities than the three smaller bears that frequently used the railway (11.9 ± 3.4 vs. 4.8 ± 0.4 ungulate carcasses/km²). The remaining nine bears, of which eight used the rail at least once, did not exhibit evidence of ingesting grain or sulfur pellets but did forage for rail-associated plants (53 ± 20% of scats), ungulates (16.4 ± 26.6%), and ants (36.7 ± 14.1%).

## Discussion

The nascent field of railway ecology is limited by a lack of information on the relative preference for attractants that promote use of railway systems by wildlife species. In this study, we examined whether a threatened population of grizzly bears in Banff and Yoho National Parks are attracted to the Canadian Pacific Railway for the unique foraging opportunities it provides. Evidence derived from grizzly bear scats, hair, and GPS data suggest that bears seek out several food attractants on the railway and their use varies among individual bears. Bears that used the rail frequently foraged for grains when they were available, however, interestingly, we found that these bears had a more species-rich diet of plants and animals than conspecifics. We found no correlation between rail use and the nitrogen and sulfur stable isotope values measured in bear hair, which might have been expected if bears consistently foraged for a particular food with a unique isotopic signature near the railway. The consumption of grain increased in fall (relative to spring and summer) and we found scats containing these products over 7 km from the rail, suggesting that bears can transport human-derived nutrients and seeds to the surrounding landscape. Rather than uniform attraction by many animals to a few types of grain, it appears that a minority of animals (4 of 18 to which we could assign scats) made extensive use of the rail (>20% of GPS-monitored days spent on the rail), where they foraging for a variety of anthropogenic and native foods depending on their body size.

The individual bears that used the railway corridor may have benefited from the unique foraging opportunities it provided. The three most common types of grain we detected in bear scats were rich in macronutrients that are important for large omnivores, including wheat and barley (high in carbohydrates), canola seeds (high in lipids), and lentils (high in crude protein; [52]). These agricultural products were more prevalent in some bear scats in the fall compared to the summer likely because grain shipments, and thus spillage, typically increase in September following the fall harvest [53]. Our results may have underestimated bear use of grain in the early spring, however, as we did not collect scats in March and April. In addition to being more readily available, bears likely seek out these high-quality foods (e.g., canola) more actively during fall hyperphagia when fewer high caloric alternate foods are available to meet the nutrient requirements required to survive the winter [49,54]. The viability of agricultural seeds after deposition by bears may also merit some attention, given the large distances (7 km) they occurred from the rail, the presence of genetically-modified products (e.g., canola), and potential for invasive spread in a protected area.

Anthropogenic food subsidies have shifted the distributions and movements of wildlife in many contexts [55]. Such human-derived food sources can cause human-wildlife conflict (reviewed by [56]) and endanger declining or sensitive populations [57]. Our data suggest that for some individual grizzly bears and particularly in fall, spilled grains are consumed along a railway where animals may be struck by passing trains. Several other species incur frequent mortalities from train collisions, including Asian elephants in India (*Elephas maximus*; [58]), European brown bears in Eastern Europe [12], and Mongolian gazelles in China (*Procapra gutturosa*; [5]). Although the causes of train-induced mortalities have not been examined extensively for these species, attraction to spilled grain may contribute to these documented mortalities. More studies are necessary to determine the relative attraction of omnivores to different agricultural products, rail-associated vegetation, dead animals, and the rail as a travel corridor.

Particular attention to the association between rail-associated attractants and wildlife mortality may be warranted for species, like grizzly bears, that exhibit strong interspecific competition and life history strategies, such as hibernation, that increase demands for efficient foraging during hyperphagia. These associations may also be especially important when these species occur in locations where native forage is limited by habitat degradation, latitude, or elevation. Grizzly bears in our study system are considered to be protein-limited [25,44], but they may also target foods rich in lipids [26]. Bears might be attracted to the rail for protein sources that include rail-killed ungulates, ants, and some agricultural products, such as lentils. Although we could not distinguish between selective and opportunistic foraging on grain, future studies could compare the relative abundance of different grains in shipments and scats. In contrast to grain, bears consumed ants and ungulates on the rail more frequently during the summer months, likely because of increased abundance of ants [59] and perhaps naïve juvenile ungulates [60]. Lipid-rich food sources that are associated with the rail include larvae and canola seeds, which are more likely to occur in summer and fall, respectively. The contrasting seasonal patterns in foraging by bears on the rail suggest that bears may use the rail for different purposes, thereby requiring a different mitigation strategy each season to reduce the risk of being killed by trains.

More detailed study of nutrition, digestibility, and plant distribution would be needed to determine the degree to which rail-associated plants are selected and consumed by bears and other species killed by trains. For example, we expected to find higher frequencies of foods in scats that are preferred by grizzly bears and readily grow along the railway, including dandelion and sweet vetch [49]. Plant parts in scats may have been more uniformly distributed because of the longer gut transit times of plants compared to some grains that pass largely undigested into scat (unpublished data; [61]). These plants are also prevalent in disturbed sites (such as ski hills) that are distant from the rail, but also attractive to bears, which may have diluted the effect of distance to rail.

We found no correlation between stable isotope values and rail use likely because only a few bears used the rail frequently, individual bears varied in their use of different attractants, and sulfur pellets are likely consumed accidentally with grain. We urge researchers, however, to identify such biomarkers in future studies. Measuring the relative use of transportation corridors by wildlife using non-invasive methods such as hair-snaring could be used to predict the number of individuals in a population at risk of being killed. Measuring mortality risk using such a method would be useful for evaluating mitigation efforts to reduce wildlife mortalities on the railway in Banff and Yoho National Parks and other transportation systems.

We did not find evidence that a majority of the bears in our study population make regular use of the railway, but instead we found an interesting bimodal pattern of rail use among the bears that used the rail. Three of the four bears that used the rail extensively were small subadults (two males and one female) and one was the largest adult male in the population. These individuals were likely in need of high-quality foods, such as grains, for either gaining or maintaining mass [49]. The positive relationship we found between mass and *δ*^15^N values in male bears indicate that the large dominant male may have fed more extensively on ungulates through direct predation ungulates and especially neonates [62] and by scavenging and out-competing other bears and carnivores at carcasses from animals that were killed by other predators, vehicles, or trains. Individual tendencies to patrol (to defend) the rail for animal carcasses may have arose out of need (e.g. body size), experience [63], or learning [64,65]. While all bears that use the rail incur risk of mortality, our data suggests that some individuals make much greater use of the rail than most animals in the population, likely increasing their risk of being killed by trains. Anecdotally, our results suggest that these animals are either young, naïve and vulnerable, or highly experienced and necessarily adept at detecting and avoiding trains. An intriguing avenue for future research would be to assess how dominant males, such as the one in this study that occupied 64 km or 49% of the railway, might influence rail use and thus mortality risk of females and subordinate males.

Our results support approaches to mitigating the risk of train-caused mortality for wildlife that reduce the availability of anthropogenic attractants. To prevent the spill of agricultural products, hopper cars should be maintained and protocols should ensure proper loading and operation [13,18]. A concurrent study detected spilled grain at all sampling sites along the railway and in all seasons [66], but spill volumes where highly variable over space and time and not readily predicted by environmental variables. The authors speculated that this pattern likely resulted from higher rates of spillage from a few leaky cars that could be identified and targeted for repair. Such proactive measures are especially important for train lines that enter designated wildlife habitat (e.g. elephants in Rajaji National Park, [67]) in seasons when natural forage is less available (our data) and at sites where mortality rates are unusually high [68]. Visible spills of grain should be removed (e.g. using vacuum equipment), rather than dispersed from the rail bed (e.g., using blowers). Similarly, carcasses should be removed as promptly as possible, with extra resources assigned to seasonal pulses in ungulate collisions on roads [69] and obligatory reporting of strikes by train operators. While the presence of spilled grain appeared to attract grizzly bears to spend more time on the railway, likely increasing their risk of collisions, it is important to consider the other factors when assessing risk such as location of rail use, local habitat, and weather.

Although we found limited support that bears used the rail to forage for rail-associated plants, removing vegetation along railways in Norway reduced moose rail-caused mortality by 56% [10] and could yield similar results in Banff. Removing vegetation along the railway could potentially reduce the activity levels of bears that use the rail to forage while also reducing the number of ungulates killed by trains. Other mitigation strategies, such as designing deterrent and warning systems to alert wildlife to the presence of trains (e.g. [70]) and reducing train speed at high-risk locations, will also be more effective with a more complete understanding of why wildlife are attracted to railways. As for other sources of human-bear conflict, the most successful approach is likely one that targets the sites and times where human-wildlife conflict or wildlife mortality are more likely to occur. Mitigation strategies that incorporate wildlife use of attractants to reduce the attractiveness of railways are crucial for preventing train collisions for many species of wildlife around the world.

## Supporting information

S1 Table Raw data of grizzly bear scat analysis. Excel table containing the dates, locations, distance to rail, and percent volumes of diet items for 230 grizzly bear scats collected in Banff and Yoho National Parks between 2012 and 2014.
(XLSX)
